# The New Isolated Building at the "Oresund Hospital," Copenhagen

**Published:** 1892-10-08

**Authors:** 


### HOSPITAL CONSTRUCTION.

### THE NEW ISOLATED BUILDING AT THE "ORESUND HOSPITAL," COPENHAGEN.

In the spring of 1891 it was agreed that a new building intended for patients suffering from an infectious disease, and which were to be kept entirely isolated, should be erected by the Corporation of Copenhagen. It was decided to place the new building as near the sea as possible, and a site belonging to the Oresund Hospital was chosen. This site has by a high hoarding been completely separated from the rest of the hospital, and the entrance is also by a separate road.

The building was taken in hand in the course of the summer, and it is now completed. It is 72 feet long and 30 feet broad; it is built of brick and roofed with slate. It has been divided into two independent, and from each other entirely separated sections, which makes it possible to use it simultaneously for persons suffering from two different diseases. In each section there are two sick rooms for respectively one and two beds, room for the sick nurse, kitchen, bath-room, lavatory, &c., all on the ground floor; and on the first floor there is accommodation for the servants.

All the rooms, both walls and ceilings, are covered with Robinson's cement, which makes a thorough and complete disinfection possible. The floors on the ground floor are terrazzo floors; on the first floors principally ordinary cement floors. The installation of gas and water and the water-heating appliances are as perfect as possible, and all the arrangements are thoroughly efficient. The board partition between this new building and the rest of the hospital is ten feet high; the partition between the two sections is a fire-proof wall. Peat litter closets have been adopted, and all sharp corners have been avoided so as to make the disinfection more complete. A pretty garden will be laid out round the house.

The hospital in question, which is one of the smaller ones, has now at its disposal a site of about ten acres, where, so far, the following buildings have been erected: One brick building, three wooden buildings, one building for observations, and the new building just described. There are altogether about 150 beds, besides a tent with 12 beds. In case of emergency the number of beds can be increased to about 200. The whole concern is pleasantly appointed and well arranged. There is a line of railway down to the landing-stage, and a special carriage, so that the patients without much trouble can be brought from a ship to the hospital. The hospital has its own steam laundry and its own disinfecting establishment.

### THE VICTORIA HOSPITAL FOR CHILDREN.

The Secretary writes that in last week's article on "Special Hospitals," page 13, we "do not seem to have taken into account the beds at the Convalescent Home always occupied. On page 31 of the report it is stated that the beds at the hospital were 86, including 12 at Margate. The number of beds, 120, quoted by you do not refer to last year's report." We gladly publish this correction, although the figures in question, "120 beds for occupation," were taken from the statement published by the Metropolitan Hospital Sunday Fund as they received it from the hospital authorities.

